# American Quackery

**Published:** 1869-10

**Authors:** 


### The Bistoury.

ELMIRA, N. Y., OCT., 1869.

The BISTOURY is published Quarterly, upon the 1st of April, July, October and January, at Fifty Cents a year in advance.

Club Rates :—$25, will be paid for every 100 subscribers.

### AMERICAN QUACKERY.

### HOW THE SICK ARE IMPOSED UPON—INGENIOUS SWINDLERS !

There is no science that furnishes so wide a field for humbuggery and rascality as that of medicine. The country is infested with innumerable self-styled "doctors"—men who cannot frame a grammatical sentence, much less advance a medical idea or comprehend a physiological truth, yet possess the tact of imposing their abominable nostrums and deplorable ignorance upon the community to a degree that is appalling.

It is said, and we believe with much truthfulness, that the American people, more than any other, court humbuggery and are more susceptible to imposition. It is certainly evident that they give support to a class of charlatans that would not be tolerated in any other country, laying claim to intelligence and refinement.

For instance, we have our traveling doctors—fellows who can be consulted at the principal hotel in every village upon specified days of the month, where they will be prepared to perform miraculous cures upon all manner of invalids, and set at defiance every imaginable form of disease. They fortify themselves behind innumerable certificates of cure, purporting to come from ministers, doctors and judges, fee the daily journals well for puffing them into notoriety—wear fine clothes—drive fast horses—and thrust their worthless physic into the faces of those credulous enough to buy. Now, it should be plain to any but the most obtuse intellect that a physician can not prescribe, with any degree of success, for a chronic ailment by seeing it once a month; and that one possessed of the skill which these pretenders accredit to themselves, would find abundant employment at home, without placing themselves at the inconvenience and expense of "drumming up" their patients abroad.

Then, we have those wonderful laying-on-of-hands chaps, who practice their tricks of legerdemain upon the "faithful" throughout the country. The only wonder is that they find human beings sufficiently low in the scale of animated nature who are willing to pay them money for their nonsensical manipulations. But, by practising upon the ignorant and superstitious, they induce them to believe that they have relieved them of some imaginary disease, and immediately securing a "certificate of cure," succeed, through its publication, in entrapping a class of people from whom we would expect better things.

Next, we have the old "cancer doctor," a prying, insinuating sort of chap, who invades your household, determined at all hazard, to sell you his "corn and cancer salve." He can cure a "rose" or a "spider" cancer in a jiffy, and we defy you to exhibit to him any sort of an excresence, or even a harmless mole or other discoloration of the skin, that he will not pronounce a cancer of the most virulent form; and if he can succeed in frightening you into the belief that you will die in twenty-four hours unless you allow him to apply his all-healing plaster, the better it will be for his pocket, leaving you much worse for your unfortunate blunder.

It is queer to observe the different forms that quackery will take upon itself. Recently we had a band of musicians make their debut among us, with gaily caprisoned horses and lavishly painted chariot, upon the sides of which was emblazoned mystic characters, announcing "Wizard Oil," for the cure of more diseases than we have space to enumerate. Between the pieces by the band, an exceedingly ingenious Yankee made his appearance, who related to the waiting crowd the marvelous cures he had performed by means of the Wizard Oil. His use of medical terms was exceedingly amusing, while his language would have done credit to a Hottentot; yet this man styled himself "Doctor," and absolutely sold hundreds of dollars worth of his nostrum to the waiting, gaping crowd; and among the purchasers we noted several citizens to whom we were in the habit of accrediting some degree of intelligence.

Then, we have a class of advertising villains who thrive and grow rich by imposing upon the unsuspecting. You will see their cards in almost every newspaper of the land—they advertise to send *free of charge,* prescriptions for curing consumption, seminal-weakness, and the like—their "only object being to benefit the afflicted." Benevolent souls! Did it ever occur to you, gentle reader, as being a little singular that these self-sacrificing, *reverend* gentlemen are able to expend thousands of dollars yearly, for the privilege of advertising their exceedingly benevolent desire of supplying you gratuitously, with their marvelous receipts? And has the fact ever entered your brain that men are not apt to incur such an expense without seeing very clearly the means by which it is to be returned to them increased many fold? If such practical thoughts have never presented themselves to you, be assured that they are facts, and that you are expected to pay liberally for the advice (?) sent you. How? you ask. We'll tell you: Send to any of those "Reverend" scoundrels who are located in New York—Rev. Martin Dutton, Rev. Edward A. Wilson, and others of the ilk—you will receive, *free*—mind you—a recipe for the cure of your malady. Now, present it to a druggist, and you will find that he is unable to supply you with half the ingredients. What will you do? Precisely what these rascals supposed you would do—inclose them $5 for the remedy already prepared! Do you see?

Are you deaf? Send to another remarkably charitable individual who styles herself Mrs. M. C. Leggett, and is domiciled somewhere in Hoboken, N. J. You will receive her "receipt for making the Indian Deafness Remedy"—we will furnish it, and save you trouble. It reads as follows:—Oil of Dippel, Oil of Scorpiôn, Oil of Chaborti, Seed of Prairie Wort—when you find the ingredients, let us know, we'll then tell you how to apply them. But, should you fail in finding them—as you certainly will—why, Mrs. L. can be induced to supply you at the nominal figure of $5—cheap, you see!

Sayre & Co., of New York, are recent candidates for your confidence and money. They will send you a sample of their wonderful consumption remedy *free*—send, get some, do! You will receive a small red looking powder by return mail, with a request to try it and send $3 for more. If you fail to do so, in the course of a month another beautifully lithographed letter will reach you, urging you to send for the remedy and regretting "that pecuniary affairs are such that we cannot send a large box of the remedy free." Doubtless, you will finally send them the coveted $3—just for their pretty little letter, so kindly written you.

Another very ingeniously contrived plan for the extortion of money, is that practised upon our young men, who, through certain disgusting practices, render themselves sickly and miserable, and only too anxious for medical advice This counsel will not be sought from the regular medical adviser of the family, as it should be, but from a sense of shame of the nature of their malady, they seek aid from a distance in order that their friends may know nothing of their troubles. The knowledge of this fact is availed of by "sharpers" in every city, who furnish such young men with miserably printed trash, bearing the attractive titles of "Lectures to Young Men"—"Errors of Youth"—"Man hood how Lost, how Restored"—"Marriage Guide," &c., all of which are simply advertisements advising the parties addressed to forward money to the authors for "remedies" that are as worthless as their pamphlets. Thousands of young men, all over our country, are entrapped by the fair promises contained in these decoy publications, and are induced to send their hard earned money to these ingenious sharks. Young men—those of you who are in need of such advice as is claimed to be given in works like those mentioned—go to your regular family physician, who will give you proper instruction, and who, if he be an honest man, will not betray your secret, thus rendering the patronage of scheming vagabonds unnecessary.

We have a precious lot of pamphlets, circulars and recipes, in our possession which we purpose giving our readers in regular allopathic doses hereafter—we hope they will be greatly benefited by those already administered.

